# Estimation of delay-adjusted all-cause excess mortality in the USA: March–December 2020

**DOI:** 10.1017/S0950268821001527

**Published:** 2021-07-02

**Authors:** Andrei R. Akhmetzhanov

**Affiliations:** Global Health Program & Institute of Epidemiology and Preventive Medicine, College of Public Health, National Taiwan University, Taipei, Taiwan

**Keywords:** COVID-19, excess mortality, pandemic, reporting delay

## Abstract

We estimate the delay-adjusted all-cause excess deaths across 53 US jurisdictions. Using provisional data collected from September through December 2020, we first identify a common mean reporting delay of 2.8 weeks, whereas four jurisdictions have prolonged reporting delays compared to the others: Connecticut (mean 5.8 weeks), North Carolina (mean 10.4 weeks), Puerto Rico (mean 4.7 weeks) and West Virginia (mean 5.5 weeks). After adjusting for reporting delays, we estimate the percent change in all-cause excess mortality from March to December 2020 with range from 0.2 to 3.6 in Hawaii to 58.4 to 62.4 in New York City. Comparing the March–December with September–December 2020 periods, the highest increases in excess mortality are observed in South Dakota (36.9–54.0), North Dakota (33.9–50.7) and Missouri (27.8–33.9). Our findings indicate that analysis of provisional data requires caution in interpreting the death counts in recent weeks, while one needs also to account for heterogeneity in reporting delays of excess deaths among US jurisdictions.

## Introduction

Estimation of the real burden imposed by the coronavirus disease-2019 (COVID-19) pandemic in its first year has been challenged by numerous factors including limited testing, the large fraction of asymptomatic or subclinical cases, and questions surrounding whether deceased individuals died of COVID-19 as the primary cause or as one of several contributing conditions [1–3]. Under these circumstances, analysis of excess mortality data represents one way to assess the actual impact of the pandemic on society [4]. However, because data on excess mortality are provisionally released, reported counts are subject to reporting delays. The lengths of reporting delays are often unclear, and strategies to adjust provisional excess mortality data to account for delays would be helpful to study the impact of the COVID-19 pandemic in real time.

The United States Centers for Disease Control and Prevention (US CDC) releases provisional death counts by week and US jurisdiction on a weekly basis [5]. Incomplete counts in the weeks preceding the publication week are caused by various factors, including administrative and processing time lags, time of year, decedent age and cause of death. According to the National Center for Health Statistics, approximately 80% of deaths are automatically processed by a system, while 20% require manual input. In view of the ongoing COVID-19 pandemic, deaths can take even longer to process. Although the completeness of the data cannot be determined directly, the associated reporting delays can be estimated using well-developed techniques [6].

The importance of reporting delays in real-time analysis of infectious disease outbreaks has been previously recognised [7–14]. In some instances, detailed characterisation of reporting delays was hindered by limited available data, for instance during outbreaks in regions with ongoing armed conflicts [11] or among refugee populations [12, 13]. In other instances, the more detailed available data allowed analysis of time-varying trends in reporting delays using *P-*splines [14] or moving time windows [15]. Both methods can be significantly hampered when only a small fraction of cases is reported, making follow-up inference of reporting delays challenging [15].

Among the published studies on excess mortality in 2020 during the COVID-19 pandemic [16–21], few adjusted their estimates for reporting delays. Kawashima and colleagues [20] conducted such an adjustment for monthly all-cause deaths in Japan based on prompt vital statistics. By contrast, Weinberger and colleagues [21] analysed more granular data consisting of weekly counts across US jurisdictions and conducted nowcasting of deaths within a Bayesian framework [15]. Their conclusions were that reporting delays significantly differed across US jurisdictions, and that excess mortality was modestly undercounted in recent weeks unless adjustment was done. Although the official CDC report acknowledged this issue [5], there is still no detailed information on differences in reporting delays between jurisdictions as well as follow-up estimation of a common shared mean reporting delay.

In the current study, we fill this gap by explicitly characterising differences in reporting delays between jurisdictions. We also provide estimates of excess mortality at the subnational level in the USA for two timeframes: from March to December and from September to December 2020. The first timeframe of our analysis covers the whole period of the pandemic in the USA in 2020. The second timeframe was chosen to encompass the timeline of the second wave of the COVID-19 pandemic.

## Methods

### Data

Provisional death counts for 2019–2020 were regularly published on the CDC website (https://www.cdc.gov/nchs/covid19/covid-19-mortality-data-files.htm) on Wednesdays at 5 p.m. Reported deaths were categorised by *Morbidity and Mortality Weekly Report* (MMWR) week of publication and by US jurisdiction where the death occurred. For the current study, 22 snapshots were collected with publication dates between September 2020 and the first week of February 2021. One snapshot published the week of 16 September 2020 (MMWR week 38) was omitted for technical reasons, which was not critical for the study. The time period containing the most recent week with non-zero deaths covered MMWR week 34 (week ending date: 22 August 2020) in the earliest collected snapshot through week 53 of 2020 (week ending date: 2 January 2021) in the last four snapshots. The death counts for the week of publication and for the preceding week as well as for the weeks of 2021 were likely to be missed in any given snapshot because of zero reported counts; all non-zero death counts less than 10 were masked by the CDC for privacy reasons. The reporting jurisdictions included the 50 states with New York state separated into two jurisdictions: New York City and the rest of the state. Additionally, the District of Columbia and Puerto Rico were among the total 53 jurisdictions.

Historical records of weekly deaths from 2014 to 2018 were retrieved from the same source as the provisional counts for 2019–2020. The structure of the dataset was analogous except that it was not subject to any changes in the future. The jurisdictional counts of reported COVID-19 deaths were assessed via the daily trends published by the CDC (https://covid.cdc.gov/covid-data-tracker/#trends_dailytrendscases (accessed 26 February 2021)).

### Reporting delay: parametric estimation (independent and partial pool model)

The reporting delay distribution describes the distribution of time periods between the occurrence of an event and its reporting to the system. The probability distribution function of the reporting delay is usually modelled using one of three unimodal distributions with positive support (*f*_*i*_(○; *θ*),  *i* = 1, 2, 3): the gamma, Weibull or lognormal distributions. The set *θ* consists of two parameters: the mean and the standard deviation (s.d.) of the reporting delay distribution.

To estimate the reporting delay, the death count 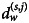 reported on week *w* by jurisdiction *j* in any of the earlier snapshots *s* = 1, …, (*S* − 1) was compared with the death count 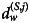 reported in the latest snapshot *S* [11, 12]. Poisson likelihood was used to infer the unknown parameters *θ*_*j*_:
1
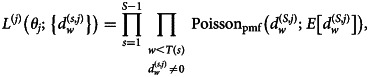
2
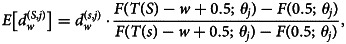
for any *w*, *j* and *s* = 1, …, (*S* − 1). The second equation accounts for the continuity factor [6]. Here, *T*(*s*) denotes the publishing times of the snapshots *s*, and Poisson_pmf_(*d*;*E*[*d*]) is the probability mass function:
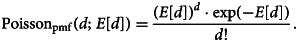
To estimate variation in reporting delays across jurisdictions, two different approaches were employed. In the first approach, the reporting delay for each jurisdiction was estimated independently, such that each likelihood *L*^(*j*)^ (*j* = 1, …, 53) was maximised with respect to *θ*_*j*_. In the second approach, a partial pool model was used to infer the common shared mean reporting delay and its s.d. [22–24]. In the latter context, the reporting delays for various jurisdictions were closely related to each other, sharing a common mean value *μ*. Any deviations from the shared value of the mean were modelled using a Student's *t*-distribution:3
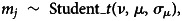
where the other two parameters were the degree of freedom *ν* and the standard error of the mean 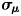. The Student's *t*-distribution (3) was chosen over the normal distribution because it is less sensitive to outliers. Like the nonparametric estimation described below, the first approach showed promise when used to nowcast the number of deaths that have yet to be reported. The second approach was used to identify the common mean *μ*, and the corresponding *P*-values (percentiles of the Student's *t*-distribution) for detecting outliers (i.e. jurisdictions significantly deviating from others in their reporting delays).

A negative binomial distribution could have been used instead of the Poisson distribution in equation (1). However, simulations showed that the value of the overdispersion parameter in the negative binomial distribution approached an arbitrarily large value, implying equivalence of the negative binomial and Poisson likelihood functions. A similar conclusion was reached in another relevant study [15].

### Mixture model

Although the reporting delay distribution was chosen from one of three unimodal distributions, this selection induced a constraint to the modelling framework by imposing a structural prior. Another approach to account for all three distributions within a single model is to consider mixtures of distributions. This strategy provides a greater degree of flexibility because each distribution contributes to the total likelihood proportionally to the relative weights *π*_*i*_ (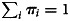), subject to the data fit. By contrast with the common practice in formulating mixture models, where each component distribution has its own set of parameters (e.g. each of the three distributions would have their own means and s.d.s), we assumed that all distributions shared the same set of parameters (mean and s.d.). This ensures a higher convergence probability of implemented Markov Chain Monte-Carlo simulations [25].

Alternatively, the best-fit distribution could be selected based on information criteria (e.g. the widely applicable information criterion (WAIC) or ‘leave-one-out’ information criterion (LOOIC) [24, 26]). However, integrating out unobserved (latent) variables from the model, such as the death counts masked by CDC, can be challenging [27]. The mixture model implements all three component distributions based on relative weights *π*_*i*_. In this case, there was no need to integrate out latent variables or to manually calculate likelihoods for each data point, as is required using other methods [28].

Following these assumptions, the total likelihood for the mixture model was defined as follows:
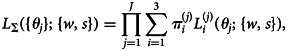
where 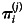 are relative weights (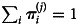) and the subscript *i* indicates one of the three distributions (*i* = 1, 2, 3). The component likelihoods 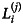 are given by equation (1) and the expected deaths 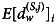 (an internal argument of the likelihoods) are given by (2) respective to each distribution *i*:4
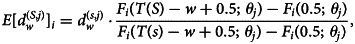
where *F*_*i*_ denotes the cumulative distribution function of the component distribution *i*. The posterior probability for each component distribution 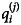 could be then determined using the equation:
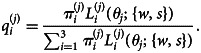


### Reporting delay: nonparametric estimation

For nonparametric estimation of the reporting delay, the reverse-time discrete hazard was defined as previously described [7, 8]: *g*_*j*_(*d*) = Pr(delay = *d* | delay ≤ *d*) = *f*_*j*_(*d*)/*F*_*j*_(*d*). Here, the variable *d* was introduced such that a zero value (*d* = 0) corresponds to the death count reported within the first 2 weeks (equivalently, within the first 10 days because all snapshots were published on Wednesdays rather than on the last day of the week). Other values (*d* = 1, 2, …, *D*) correspond to reporting delays of weeks, respectively. The upper bound *D* denotes the maximum delay, implying that *F*_*j*_(delay ≥ *D*) = 1. Finally, *g*_*j*_(0) = 1 was imposed, and other hazards *g*_*j*_(*d* > 0) were found by fitting the probability distribution functions 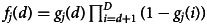 to the data. Equations (1), (4) were used, accounting for the only difference in defining the cumulative distribution functions:5
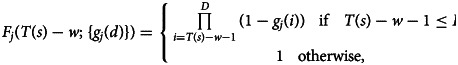
where the parameter *D* was set to 20 weeks in the simulations.

### Nowcasting procedure

To predict the number of deaths not yet reported by the surveillance system, a prospective nowcasting framework was applied [11, 29]. The number of yet unreported deaths on a given week was sampled from a negative binomial distribution that followed the failure-counting interpretation [30]. The first parameter of the negative binomial distribution (the number of ‘failures’) was the number of already reported deaths during that week, whereas the second parameter (the probability of ‘success’) was the cumulative distribution function of the reporting delay counted from week of death, *w*, to the publication date of the latest snapshot.

### Expected excess mortality

The expected weekly number of deaths was estimated using a Poisson linear regression model [21] involving a seasonal component but neglecting to adjust for severe influenza and associated pneumonia. The posterior median and the 95% upper bound were set as two thresholds. The range of differences between the nowcasted number of deaths and each of these thresholds was then reported as excess deaths as in previous studies [5, 20]. All negative differences were assigned to zero. The reader is referred to the Appendix for additional mathematical details of the statistical framework.

### Technical details

**Fig. 1.**
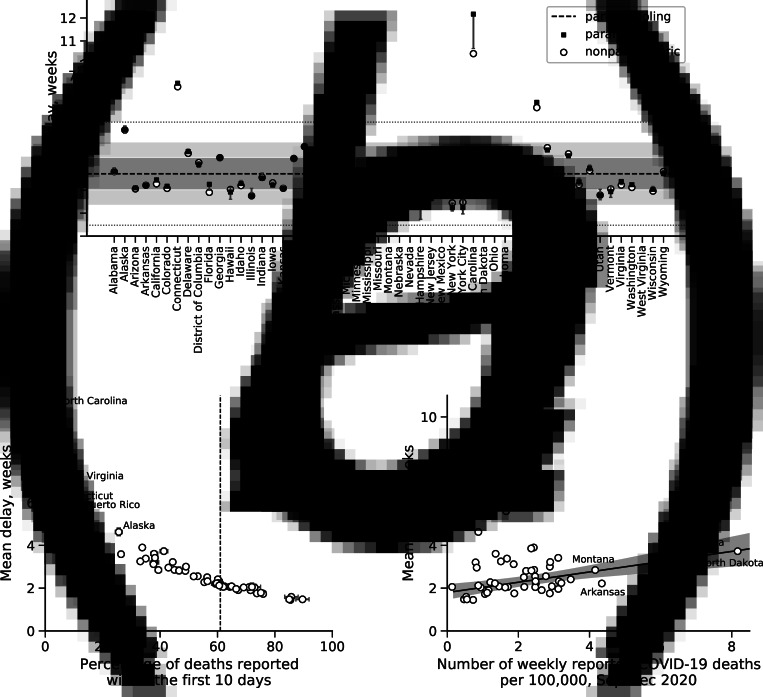
(a) Mean reporting delay by jurisdiction using different estimation approaches (legend). Error bars indicate the 95% CI for individually estimated reporting delays using a parametric model. Dashed line indicates a common mean delay inferred from the partial pool model. The entire shaded area indicates the 95% CI for the common mean delay, whereas the dark shaded area covers the interquartile range of the posterior. (b) Relationship between the fraction of deaths reported within the first 10 days and the mean reporting delay by jurisdiction obtained from non-parametric estimation of the reporting delay distribution. Dashed line indicates an estimate of 61% cited in technical notes of CDC [5]. (c) Correlation between number of reported COVID-19 deaths per 100 000 from September to December 2020 and the mean reporting delay by jurisdiction. Solid line is obtained from a linear regression model. Shaded area indicates 95% CI.

To infer individual mean reporting delays and perform nowcasting, nonparametric estimation of the reporting delay distribution was used. A parametric estimation was implemented only for verification purposes (Fig. 1). A partial pool model was used to calculate the common mean of the reporting delay shared across jurisdictions. Because of excessive computational time requirements, only a lognormal distribution was implemented in the partial pool model. The choice of the lognormal distribution was guided by its dominant selection while fitting the mixture models for various jurisdictions (Appendix Fig. 1).

Statistical inference was conducted within the Bayesian framework realised in CmdStan (version 2.26, https://mc-stan.org). Pre- and post-processing of the data and results were performed in the Python environment (version 3.8). The code snippets are available at http://github.com/aakhmetz/Excess-mortality-in-US-2020.

## Results

We started our analysis by fitting the reporting delays for all 53 US jurisdictions independently of one another. The patterns of the mean reporting delays for all-cause excess mortality suggested clustering around a common value (Fig. 1a and Appendix Fig. 1). A common shared mean delay was calculated at 2.8 weeks (95% credible interval (CI): 2.4, 3.0 weeks). Most jurisdictions (32/53, 60.4%) had mean reporting delays within the interquartile range of 2.0–3.4 weeks. All jurisdictions except for four had mean reporting delays within the 95th percentile range of 0.5–5.0 weeks. Connecticut, North Carolina, Puerto Rico and West Virginia had mean reporting delays above the 95th percentile (shown as a dotted line in Fig. 1a). Suspecting those jurisdictions to be outliers, we first identified that North Carolina clearly deviated from the other jurisdictions with a mean delay of 10.4 weeks (median *P*-value 0.001) [31]. Excluding North Carolina from the partial pool model, we determined that the other three jurisdictions also clearly deviated from the remainder: Connecticut reported death counts with a mean delay of 5.8 weeks (median *P*-value = 0.006), Puerto Rico with a mean delay of 4.7 weeks (median *P*-value = 0.028) and West Virginia with a mean delay of 5.5 weeks (median *P*-value = 0.010) (Appendix Fig. 2).

To identify jurisdictions experiencing delays in reporting not as extreme as the four jurisdictions above, we investigated the correlation between fraction of deaths reported within the first 10 days and mean reporting delays. Figure 1b shows clustering of points around the value of 61% identified earlier in the technical notes of the CDC [5]. We hypothesised that points located on the left-hand side of the corresponding dashed vertical line in Figure 1 represented jurisdictions with longer anticipated reporting delays.

Suspecting that longer delays were caused by larger numbers of reported COVID-19 cases in jurisdictions, we assessed the association between mean reporting delay and cumulative number of reported COVID-19 deaths per 100 000 from September to December 2020. We first compared a linear regression model with a non-zero slope and Student's *t*-distribution to minimise the effect of outliers with a null model with a zero slope. Following an LOOIC, the null model was rejected (ΔLOOIC = 10.1; relative weight for alternative model: 0.93). The alternative model predicted that an additional 4.5 reported COVID-19 deaths per week per 100 000 individuals was associated with 1 additional week in the reporting delay (Fig. 1c).

**Fig. 2.**
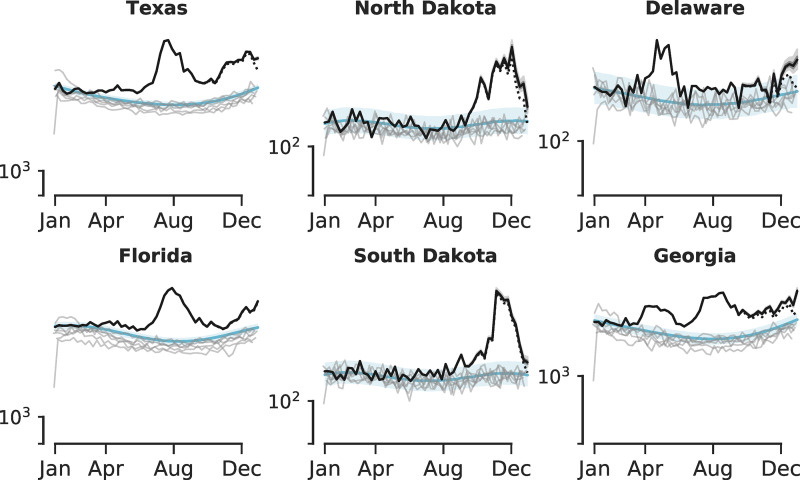
Comparing the nowcasted all-cause excess deaths by week of 2020 with expected deaths. Black line and grey shaded area show the median and 95% CI of the nowcasted death count in 2020. Blue line and blue shaded area indicate the median and 95% CI derived from posterior distributions of the expected weekly deaths. Individual grey lines indicate the reported deaths in 2014–2019.

Figure 2 shows all-cause excess mortality adjusted by the reporting delays for six jurisdictions. Among them were two jurisdictions (Texas and Florida) with the highest numbers of reported COVID-19 deaths from September to December 2020, two jurisdictions (South Dakota and North Dakota) with the highest COVID-19 deaths per 100 000 over the same period, and two jurisdictions (Delaware and Georgia) where adjustment for reporting delay led to an increase instead of a decrease in the unadjusted counts over the last 2 weeks of 2020 (*cf.* solid and dotted lines in Fig. 2). Conducting a validation procedure for nowcasting using earlier cutoff times (Appendix Fig. 3), we found, similarly to [21], that the performance for nowcasting was conservative because the nowcasted death counts are likely to be underestimates of the final counts. The values from the latest snapshot published on 11 February 2021 are expected to reflect the final counts of 2020 with greater certainty compared with prior snapshots because the time elapsed between the publication date and the last week of 2020 exceeds the estimated mean reporting delay in most jurisdictions. The results of nowcasting for all jurisdictions are shown in Appendix Figure 4.

Next, we calculated excess mortality by jurisdiction for the entire period of the COVID-19 pandemic (from March to December 2020; Table 1) and for the second wave (from September to December 2020; Appendix Table 1). As expected, adjustment did not significantly alter the estimated numbers of deaths over the entire period. The jurisdictions with the largest percent changes following adjustment were New York City with the range of 58.4–62.4% (26 212–28 040 excess deaths), New Jersey at 32.4–37.0% (19 571–22 369 excess deaths) and Texas at 23.7–27.1% (40 413–46 127 excess deaths). The jurisdictions with the smallest changes following adjustment were Hawaii at 0.2–3.6% (24–357 excess deaths), Maine at 0.2–3.8% (32–497 excess deaths) and Alaska at 0.3–7.9% (14–316 excess deaths). Comparing the September–December 2020 period with the March–December 2020 period, the jurisdictions with the largest percent changes following adjustment were South Dakota at 36.9–54.0% (1073–1568 excess deaths), North Dakota at 33.9–50.7% (906–1354 excess deaths) and Missouri at 27.8–33.9% (6196–7543 excess deaths), while the jurisdictions with the smallest changes following adjustment were Puerto Rico at 0.2–2.5% (22–271 excess deaths), Hawaii at 0.5–5.9% (22–242 excess deaths) and Maine at 0.6–6.9% (32–374 excess deaths). The provided values indicate deviations from two thresholds of the median and the 95th percentile as it was described above.

**Table 1.** Excess mortality by jurisdiction for the entire period of the COVID-19 pandemic (from March to December 2020)

Jurisdiction	Observed deaths, No.	Expected deaths, No.	Excess deaths, No.	Excess deaths, %	Observed deaths unadjusted, No.
Alabama	54 066 (53 985–54 153)	42 919	8379–11 147	19.5–26.0	53 626
Alaska	4148 (4121–4180)	4021	14–316	0.3–7.9	4033
Arizona	66 003 (65 954–66 069)	51 564	11 316–14 438	21.9–28.0	65 887
Arkansas	32 208 (32 161–32 265)	26 819	3617–5450	13.5–20.3	32 086
California	270 183 (270 093–270 316)	224 603	39 126–45 580	17.4–20.3	269 998
Colorado	40 476 (40 437–40 528)	34 588	3592–5908	10.4–17.1	40 388
Connecticut	30 188 (30 109–30 271)	25 606	4239–5641	16.6–22.0	29 419
Delaware	9224 (9189–9263)	7692	534–1556	7.0–20.2	9057
District of Columbia	6341 (6319–6367)	5294	518–1112	9.8–21.0	6282
Florida	206 478 (206 370–206 602)	177 187	23 499–29 290	13.3–16.5	206 198
Georgia	87 258 (87 142–87 378)	71 905	11 662–15 353	16.2–21.4	86 083
Hawaii	10 052 (10 024–10 086)	10 024	24–357	0.2–3.6	9978
Idaho	14 044 (14 022–14 073)	11 966	1052–2150	8.8–18.0	13 998
Illinois	110 356 (110 292–110 442)	88 734	17 459–21 622	19.7–24.4	110 215
Indiana	65 198 (65 113–65 293)	56 097	6677–9667	11.9–17.2	64 846
Iowa	30 142 (30 083–30 207)	25 249	3152–4947	12.5–19.6	29 965
Kansas	26 461 (26 421–26 511)	22 179	2705–4305	12.2–19.4	26 364
Kentucky	46 586 (46 502–46 675)	40 123	3867–6469	9.6–16.1	46 086
Louisiana	48 321 (48 232–48 414)	38 426	7171–9895	18.7–25.8	47 416
Maine	13 115 (13 090–13 149)	12 914	32–497	0.2–3.8	13 054
Maryland	51 214 (51 155–51 281)	41 140	7264–10 073	17.7–24.5	51 066
Massachusetts	58 740 (58 685–58 808)	48 725	8263–10 230	17.0–21.0	58 596
Michigan	98 484 (98 420–98 569)	81 462	13 180–17 021	16.2–20.9	98 343
Minnesota	44 283 (44 212–44 363)	38 825	3379–5525	8.7–14.2	44 027
Mississippi	33 860 (33 802–33 926)	25 799	5876–8061	22.8–31.2	33 624
Missouri	64 564 (64 450–64 680)	53 539	7989–11 032	14.9–20.6	63 734
Montana	10 241 (10 215–10 275)	8767	862–1631	9.8–18.6	10 177
Nebraska	16 840 (16 803–16 885)	13 630	1808–3220	13.3–23.6	16 739
Nevada	26 722 (26 669–26 782)	22 357	2657–4405	11.9–19.7	26 542
New Hampshire	11 527 (11 502–11 559)	10 224	388–1326	3.8–13.0	11 468
New Jersey	82 788 (82 728–82 863)	60 452	19 571–22 369	32.4–37.0	82 640
New Mexico	19 076 (19 021–19 137)	15 467	2080–3657	13.5–23.6	18 821
New York	102 033 (101 964–102 121)	82 403	16 268–19 679	19.7–23.9	101 869
New York City	72 843 (72 800–72 900)	44 910	26 212–28 040	58.4–62.4	72 739
North Carolina	65 662 (65 415–65 938)	79 594	4445–6935	5.6–8.7	63 403
North Dakota	7747 (7708–7791)	6368	906–1472	14.2–23.1	7559
Ohio	121 784 (121 654–121 921)	100 427	16 944–21 357	16.9–21.3	121 099
Oklahoma	38 322 (38 276–38 376)	32 512	3681–5818	11.3–17.9	38 111
Oregon	33 847 (33 791–33 910)	30 578	1159–3269	3.8–10.7	33 634
Pennsylvania	132 048 (131 914–132 189)	110 043	17 640–22 086	16.0–20.1	131 460
Puerto Rico	23 379 (23 327–23 433)	25 020	268–1237	1.1–4.9	23 081
Rhode Island	10 289 (10 261–10 321)	8435	913–1880	10.8–22.3	10 212
South Carolina	50 111 (50 064–50 171)	42 426	5528–8176	13.0–19.3	49 984
South Dakota	8763 (8729–8804)	6904	1095–1899	15.9–27.5	8660
Tennessee	75 068 (74 983–75 165)	63 567	8208–11 500	12.9–18.1	74 795
Texas	216 411 (216 245–216 580)	170 284	40 413–46 127	23.7–27.1	215 054
Utah	18 869 (18 839–18 908)	16 202	1248–2667	7.7–16.5	18 801
Vermont	5243 (5225–5267)	4557	125–709	2.7–15.6	5204
Virginia	67 142 (67 069–67 225)	59 084	4819–8057	8.2–13.6	66 929
Washington	52 712 (52 667–52 771)	47 614	2521–5230	5.3–11.0	52 607
West Virginia	20 715 (20 617–20 815)	18 951	599–1857	3.2–9.8	19 498
Wisconsin	53 202 (53 149–53 269)	45 756	4848–7451	10.6–16.3	53 079
Wyoming	4752 (4733–4777)	4110	213–726	5.2–17.7	4709

The numbers in parenthesis indicate the 95% CI. The range shown in two columns for the excess deaths denotes the range of differences between the nowcasted number of deaths and each of two thresholds: the 95th percentile and the median of the posterior for the expected number of deaths.

## Discussion

Adjustment of provisional all-cause excess deaths by reporting delays as currently documented on the CDC website relies on estimates obtained from provisional data for 2018–2019 [5]. Adjustment of delays using recent data from 2020 has been carried out, but not explicitly reported. Here, we quantified jurisdictional reporting delays using the latest data from the second half of 2020. According to our estimates, four jurisdictions out of 53 (Connecticut, North Carolina, Puerto Rico and West Virginia) reported excess mortality with substantial time lags that were likely related to administrative factors. On the one hand, the percentage of deaths reported within the first 10 days in those four prefectures was much smaller compared to the overall mean of 61% (Fig. 1b). On the other hand, there was no evident correlation between the mean reporting delay and the average weekly number of reported COVID-19 cases for September–December 2020 (Fig. 1c). However, longer reporting delays in some other jurisdictions such as South Dakota or North Dakota were likely be caused by the burden of the COVID-19 pandemic (Fig. 1c). We determined that an increase of approximately 4–5 reported COVID-19 deaths per 100 000 individuals per week was associated with an additional 1 week in the reporting delay. Overall, we found that jurisdictional reporting of death counts had delays of 2–3 weeks, which, nevertheless, represents a significant improvement compared with 2015–2016 [32]. In 2015–2016, 61.9% of all-cause deaths were reported within the first 5 weeks. However, the same fraction of deaths was reported within the first 10 days in 2020. Additionally, only some jurisdictions significantly deviated from that value during the second half of 2020 (Fig. 1b).

When we assessed jurisdictional all-cause excess mortality from September to December 2020, we found that Puerto Rico had the lowest estimated number, potentially because of significant delays in reporting. This result confirms the importance of accounting for reporting delays when analysing provisional death counts and performing nowcasting. Excess mortality from March to December 2020 was less affected by reporting delays; however, some underestimation of nowcasted death counts can still be observed.

From a methodological point of view, we employed several different approaches to estimate the reporting delay. Both non-parametric and parametric estimation of the reporting delay yielded similar results, confirming the validity of our methodology. The non-parametric estimation was the easiest to implement, but was prone to overfitting the data. In contrast, a partial pool model less sensitive to overfitting can be used for deriving common characteristics shared across jurisdictions [22, 23].

Our study had several limitations. First, we considered only all-cause excess mortality, and different underlying causes of death may have contributed differentially to the reporting delay. COVID-19-associated deaths may require additional post-mortem examinations, leading to longer reporting delays especially during the first year of the COVID-19 pandemic. The reporting delay can also differ based on age, race and ethnicity as described elsewhere [15, 33]. Second, the nowcasting procedure used in our study does not incorporate a time-varying trend in the reporting delay [14, 15] and does not include a random effect [7, 8]. It also considers the contributions across different snapshots and across weeks to be independent. Implementation of a nowcasting procedure incorporating these factors would require a more sophisticated approach with construction of a two-dimensional contingency matrix of number of deaths with the week of death on one margin and the reporting delay on the other margin [14]. This was not feasible for our aggregated dataset consisting of subsequently released snapshots. For example, some re-arrangements of weekly numbers were observed for weekly death counts in Vermont, which would lead to negative differences between subsequent snapshots, and thus negative elements of the contingency matrix. Furthermore, McGough and colleagues [15] also showed that nowcasting remains challenging when low reporting rates were observed (e.g. in Connecticut, North Carolina and Puerto Rico). Under these conditions, both simpler approaches such as those used in our study and the more sophisticated approaches used elsewhere [8, 14, 15] will be limited in their performance.

Our study shows necessity for adjustment of excess death counts by the reporting delay which is rather different across jurisdictions of the USA. A more detailed cause-specific and multifactorial analysis (e.g. by age, gender, ethnicity and socio-economic status) is required to further differentiate the reporting delay and allow more accurate real-time assessments of the burden of COVID-19 pandemic in the future.

## Data Availability

All data used for this study can be found at: http://github.com/aakhmetz/Excess-mortality-in-US-2020.

## Supplementary material

For supplementary material accompanying this paper visit http://dx.doi.org/10.1017/S0950268821001527. click here to view supplementary material
