# Supplementary material for: Estimation of delay-adjusted all-cause excess mortality in the USA: March–December 2020
Source: Epidemiol Infect. 2021 Jul 2;149:e156. doi: 10.1017/S0950268821001527 (PMC8296018; doi:10.1017/S0950268821001527)
Supplement: Supplementary file 1 [file S0950268821001527sup001.docx]

**Estimation of delay-adjusted all-cause excess mortality in the USA: March–December 2020**Andrei R. Akhmetzhanov

**Appendix**


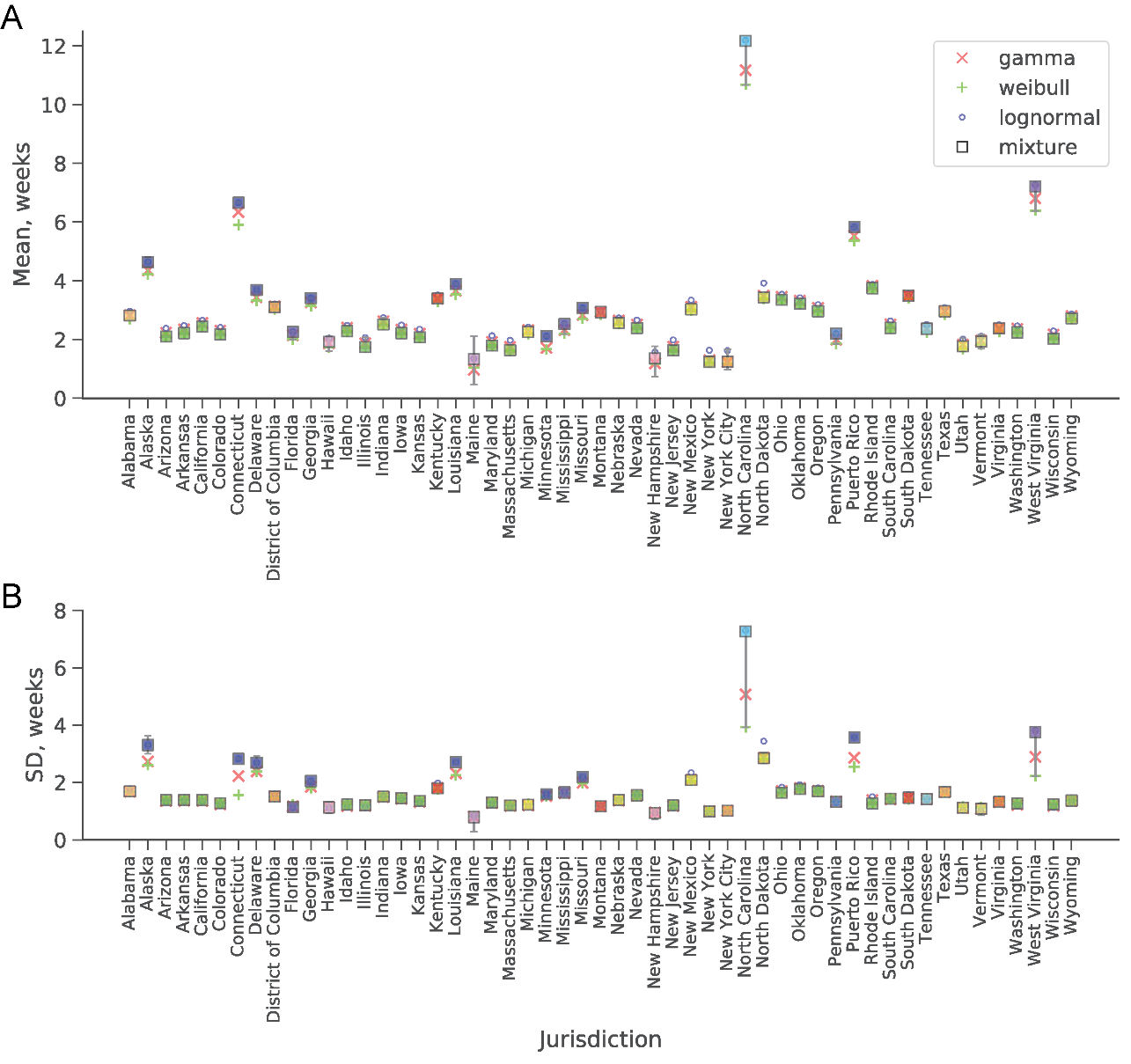


**Appendix Figure 1.** The mean and standard deviation of the reporting delay distribution estimated by one of three unimodal distributions or their mixture model (Legend). The color of the point for the mixture model indicates the relative weights of the component distributions according to the spectral color palette.





**Appendix Figure 2.** Identified *p*-values for deviations of the mean reporting delays of individual jurisdictions using the partial pool model with underlying lognormal distribution. North Carolina was excluded from the analysis. The error bar indicates 95% credible interval, while a thicker interior line segment shows the interquartile range of the posterior. The position of each point indicates the median value.


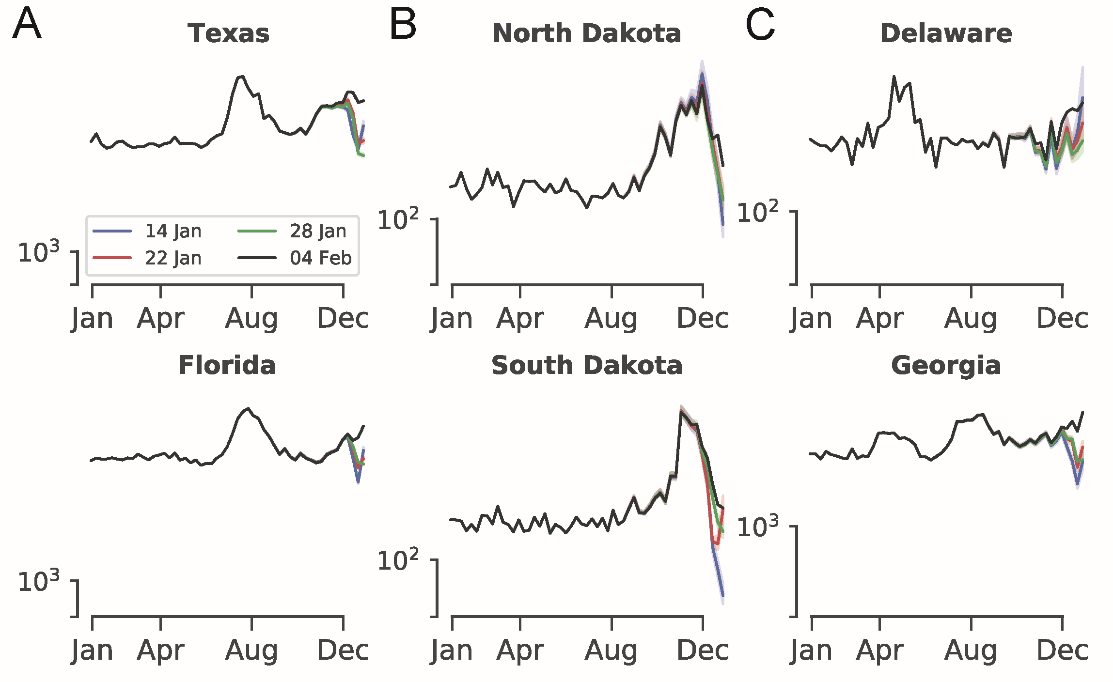


**Appendix Figure 3.** Nowcasted excess deaths with different cut-off times (Legend).


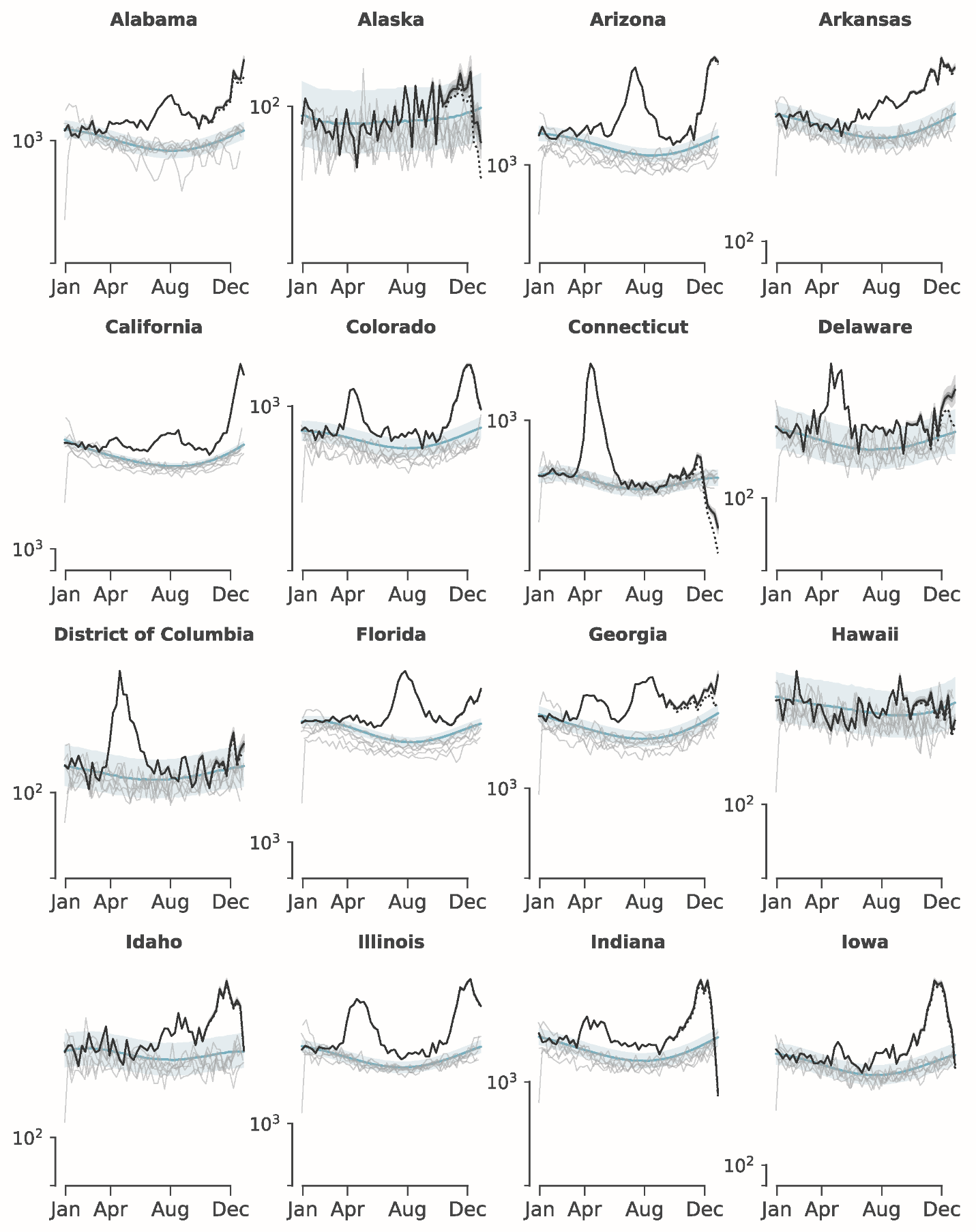


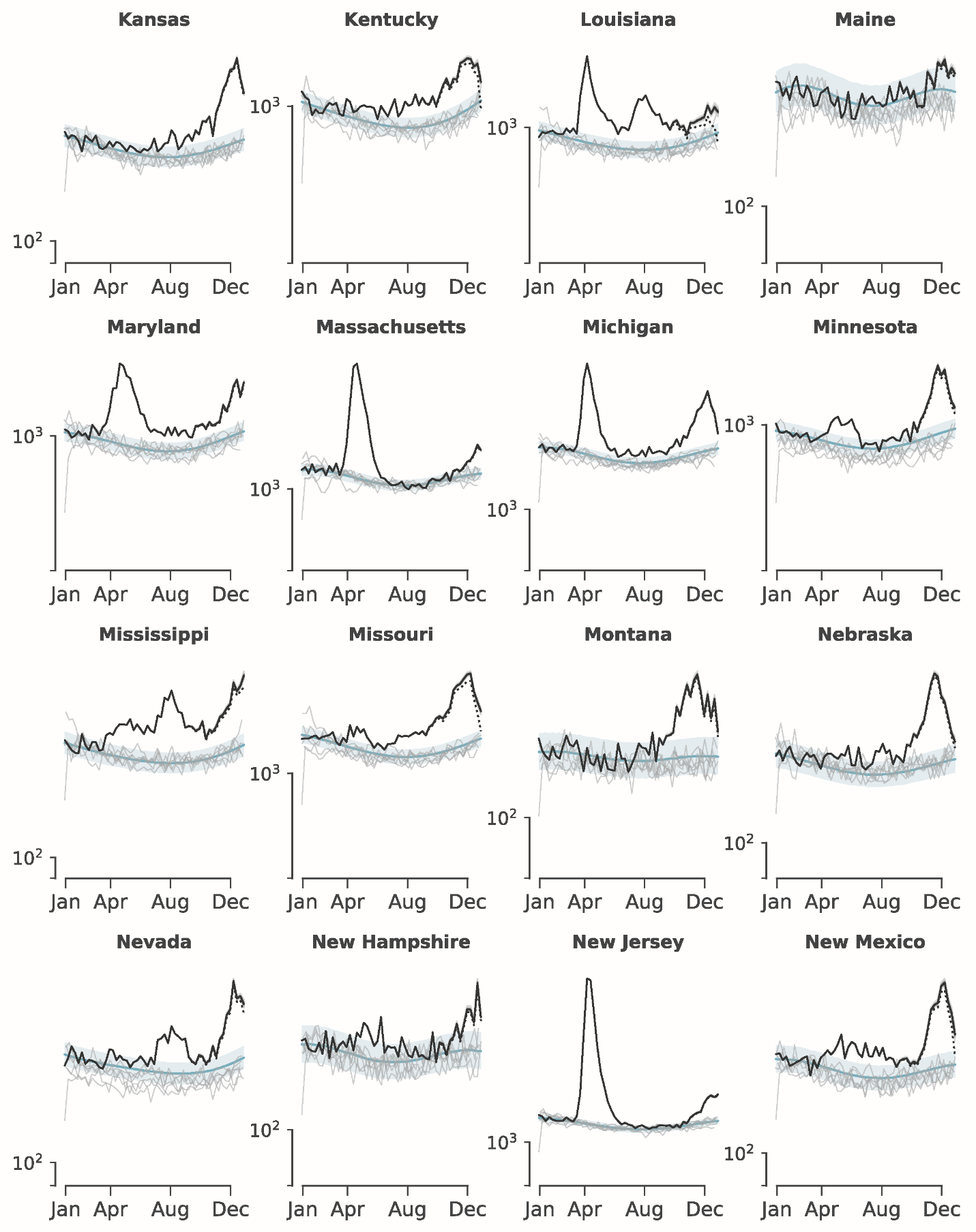


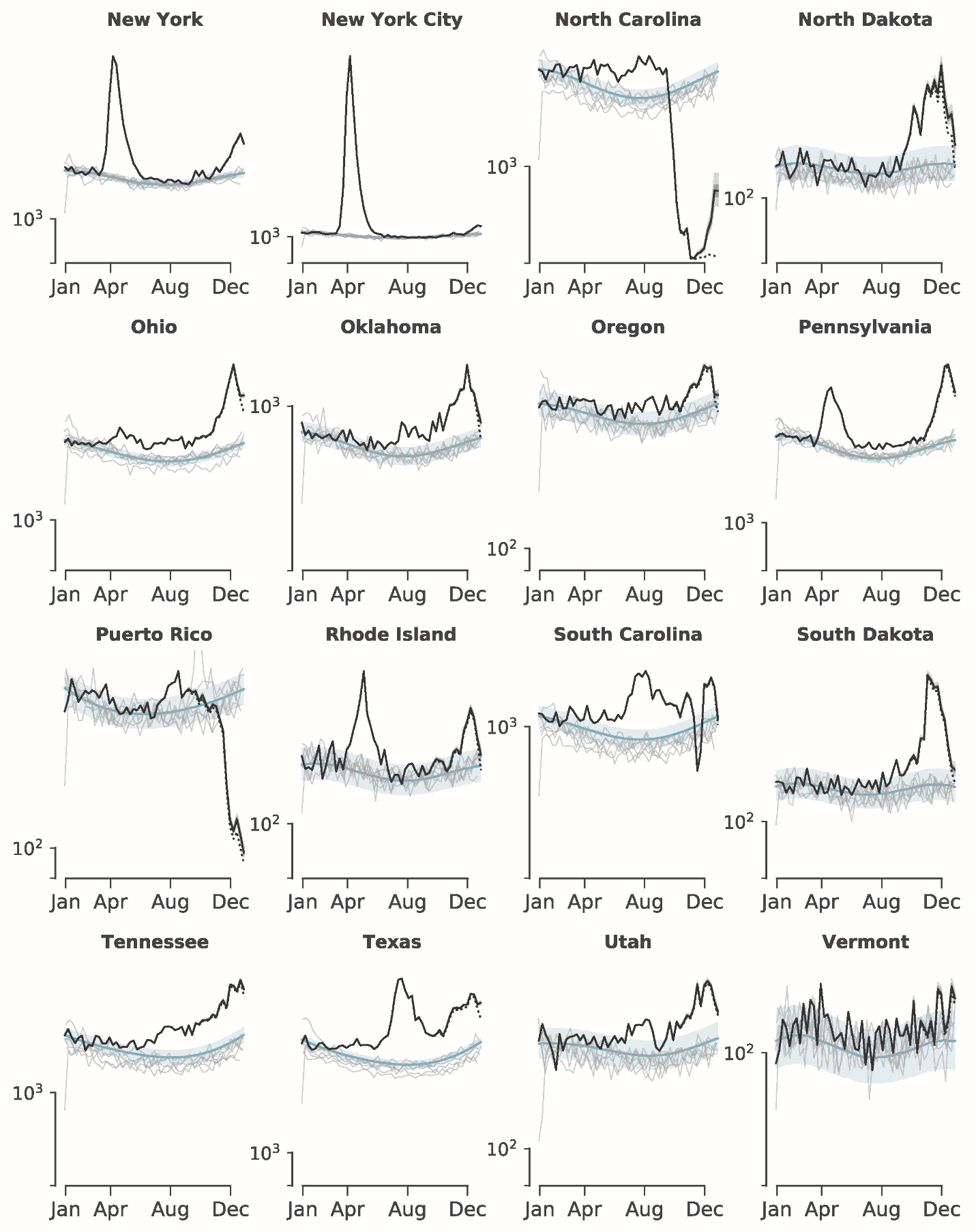


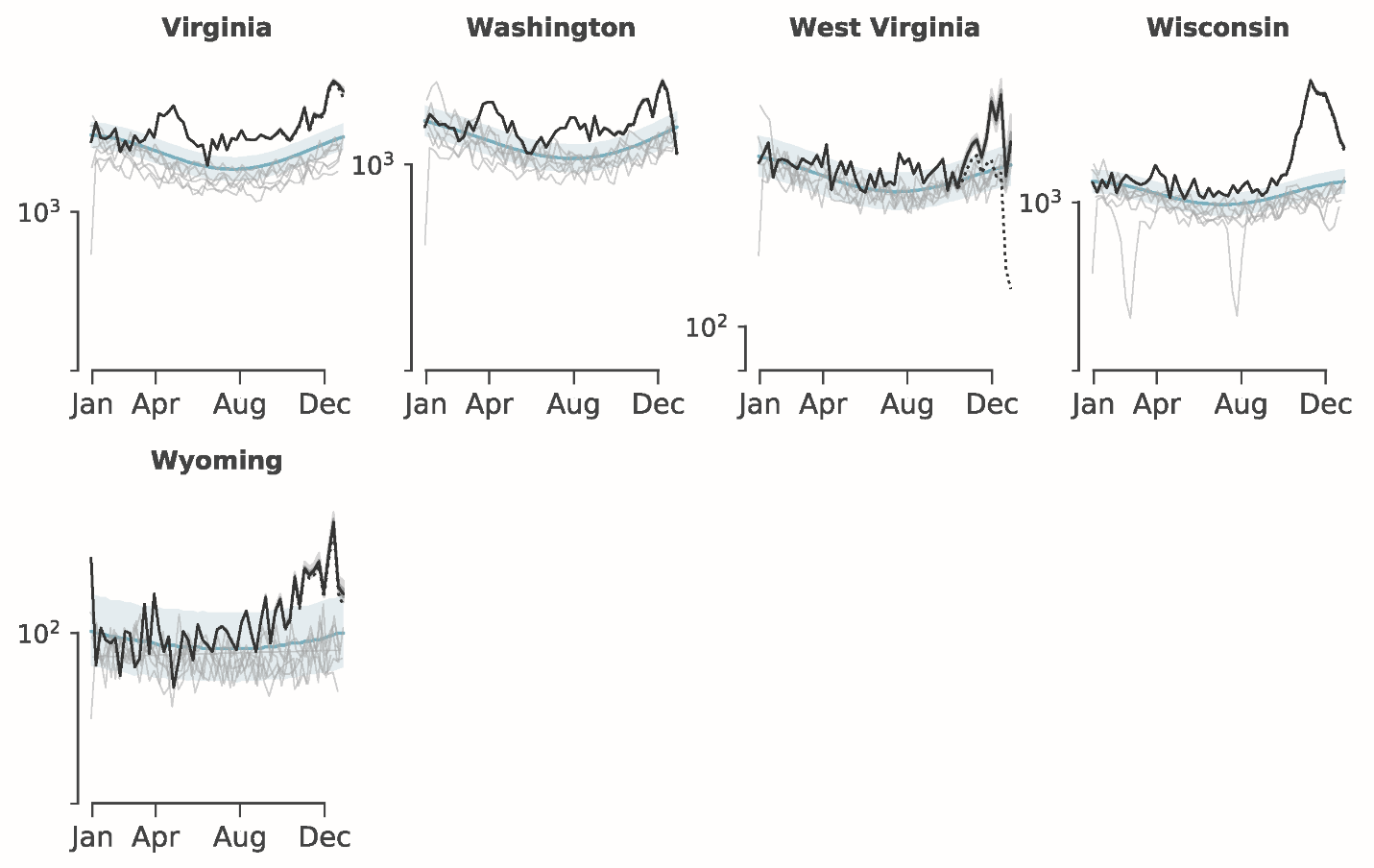


**Appendix Figure 4.** Nowcasted (black solid) and observed (dashed) excess deaths respectively to estimated expected deaths using historical data from 2014 to March 2020 and model developed by Weinberger and colleagues. Blue shaded area shows 95% credible interval, while blue solid line indicates the median values. Thin grey lines show individual counts from the historical data.

**Appendix Table 1.** Excess mortality by jurisdiction for the second wave of the COVID-19 pandemic (from September to December 2020). The numbers in parenthesis indicate the 95% credible interval. The range shown in two columns for the excess deaths denote the range of differences between the nowcasted number of deaths and each of two thresholds: the 95th percentile and the median of the posterior for the expected number of deaths.

| **Jurisdiction** | **Observed deaths, No.** | **Expected deaths, No.** | **Excess  deaths, No.** | **Excess deaths, %** | **Observed deaths unadjusted, No.** |
| --- | --- | --- | --- | --- | --- |
| Alabama | 23342  (23261, 23429) | 17838 | 4296–5504 | 24.1–30.9 | 22902 |
| Alaska | 1858  (1831, 1890) | 1688 | 12–226 | 0.7–13.4 | 1743 |
| Arizona | 26987  (26938, 27053) | 21132 | 4551–5855 | 21.5–27.7 | 26871 |
| Arkansas | 14832  (14785, 14889) | 11234 | 2643–3598 | 23.5–32.0 | 14710 |
| California | 117387  (117297, 117520) | 93433 | 21232–23954 | 22.7–25.6 | 117202 |
| Colorado | 17598  (17559, 17650) | 14437 | 2224–3170 | 15.4–22.0 | 17510 |
| Connecticut | 10426  (10347, 10509) | 10632 | 299–800 | 2.8–7.5 | 9657 |
| Delaware | 3744  (3709, 3783) | 3215 | 131–546 | 4.1–17.0 | 3577 |
| District of Columbia | 2415  (2393, 2441) | 2236 | 21–224 | 0.9–10.0 | 2356 |
| Florida | 83524  (83416, 83648) | 72802 | 8288–10722 | 11.4–14.7 | 83244 |
| Georgia | 36168  (36052, 36288) | 30135 | 4465–6033 | 14.8–20.0 | 34993 |
| Hawaii | 4225  (4197, 4259) | 4070 | 22–242 | 0.5–5.9 | 4151 |
| Idaho | 6346  (6324, 6375) | 4915 | 838–1431 | 17.0–29.1 | 6300 |
| Illinois | 47698  (47634, 47784) | 37276 | 8682–10422 | 23.3–28.0 | 47557 |
| Indiana | 28083  (27998, 28178) | 23513 | 3939–5133 | 16.8–21.8 | 27731 |
| Iowa | 13980  (13921, 14045) | 10634 | 2520–3394 | 23.7–31.9 | 13803 |
| Kansas | 12660  (12620, 12710) | 9276 | 2508–3384 | 27.0–36.5 | 12563 |
| Kentucky | 20819  (20735, 20908) | 16808 | 2834–4011 | 16.9–23.9 | 20319 |
| Louisiana | 18978  (18889, 19071) | 16017 | 1813–2961 | 11.3–18.5 | 18073 |
| Maine | 5687  (5662, 5721) | 5384 | 32–374 | 0.6–6.9 | 5626 |
| Maryland | 20866  (20807, 20933) | 17223 | 2459–3643 | 14.3–21.2 | 20718 |
| Massachusetts | 21959  (21904, 22027) | 20250 | 972–1828 | 4.8–9.0 | 21815 |
| Michigan | 41659  (41595, 41744) | 33956 | 6037–7703 | 17.8–22.7 | 41518 |
| Minnesota | 20027  (19956, 20107) | 16393 | 2574–3634 | 15.7–22.2 | 19771 |
| Mississippi | 14321  (14263, 14387) | 10743 | 2644–3578 | 24.6–33.3 | 14085 |
| Missouri | 29817  (29703, 29933) | 22274 | 6196–7543 | 27.8–33.9 | 28987 |
| Montana | 4994  (4968, 5028) | 3600 | 846–1394 | 23.5–38.7 | 4930 |
| Nebraska | 7929  (7892, 7974) | 5697 | 1547–2232 | 27.2–39.2 | 7828 |
| Nevada | 11755  (11702, 11815) | 9200 | 1701–2555 | 18.5–27.8 | 11575 |
| New Hampshire | 4884  (4859, 4916) | 4277 | 247–626 | 5.8–14.6 | 4825 |
| New Jersey | 29264  (29204, 29339) | 25135 | 3008–4162 | 12.0–16.6 | 29116 |
| New Mexico | 8463  (8408, 8524) | 6392 | 1377–2071 | 21.6–32.4 | 8208 |
| New York | 39504  (39435, 39592) | 34380 | 3810–5157 | 11.1–15.0 | 39340 |
| New York City | 20109  (20066, 20166) | 18842 | 789–1364 | 4.2–7.2 | 20005 |
| North Carolina | 13188  (12941, 13464) | 33272 | 509–771 | 1.5–2.3 | 10929 |
| North Dakota | 4024  (3985, 4068) | 2670 | 906–1354 | 33.9–50.7 | 3836 |
| Ohio | 54856  (54726, 54993) | 41908 | 11102–12948 | 26.5–30.9 | 54171 |
| Oklahoma | 17487  (17441, 17541) | 13554 | 2878–3933 | 21.2–29.0 | 17276 |
| Oregon | 14470  (14414, 14533) | 12721 | 817–1749 | 6.4–13.7 | 14257 |
| Pennsylvania | 57211  (57077, 57352) | 45914 | 9361–11297 | 20.4–24.6 | 56623 |
| Puerto Rico | 8068 (8016, 8122) | 10638 | 22–271 | 0.2–2.5 | 7770 |
| Rhode Island | 4200  (4172, 4232) | 3504 | 267–696 | 7.6–19.9 | 4123 |
| South Carolina | 20423  (20376, 20483) | 17776 | 2134–3139 | 12.0–17.7 | 20296 |
| South Dakota | 4475  (4441, 4516) | 2906 | 1073–1568 | 36.9–54.0 | 4372 |
| Tennessee | 34019  (33934, 34116) | 26573 | 5976–7445 | 22.5–28.0 | 33746 |
| Texas | 92516  (92350, 92685) | 71252 | 18861–21264 | 26.5–29.8 | 91159 |
| Utah | 8338  (8308, 8377) | 6706 | 930–1632 | 13.9–24.3 | 8270 |
| Vermont | 2235  (2217, 2259) | 1898 | 76–340 | 4.0–17.9 | 2196 |
| Virginia | 28563  (28490, 28646) | 24730 | 2410–3833 | 9.7–15.5 | 28350 |
| Washington | 22093  (22048, 22152) | 19790 | 1258–2431 | 6.4–12.3 | 21988 |
| West Virginia | 8907  (8809, 9007) | 7932 | 431–1028 | 5.4–13.0 | 7690 |
| Wisconsin | 24819  (24766, 24886) | 19319 | 4306–5499 | 22.3–28.5 | 24696 |
| Wyoming | 2255  (2236, 2280) | 1711 | 203–544 | 11.9–31.8 | 2212 |

**Appendix text**

*Nowcasting procedure*
We use non-parametric estimation for nowcasting of the weekly death counts. The nowcasted deaths are sampled from the negative binomial distribution with the mean:

|  | $d_{w}^{(S,j)}\cdot\frac{1-F_{j}\left( T\left( S \right)-w;\left\{ g_{j}\left( d \right) \right\} \right)}{F_{j}\left( T\left( S \right)-w;\left\{ g_{j}\left( d \right) \right\} \right)},$ |  |
| --- | --- | --- |

and shape $d_{w}^{(S,j)}$. This is equivalent to the failure-counting definition of the negative binomial distribution with the number of “failures” equal to $d_{w}^{(S,j)}$ and probability of “success” equal to $F_{j}\left( T\left( S \right)-w;\left\{ g_{j}\left( d \right) \right\} \right)$.

*Expected excess deaths*
To calculate expected deaths $\tilde{d}_{w}^{\left( y,j \right)}$ for calendar year $y$, week $w$, and jurisdiction $j$, we used a previously developed model of Weinberger and colleagues [S1] that follows Poisson likelihood:

|  | $\tilde{d}_{w}^{\left( y,j \right)} \sim\mathrm{Poisson}\left( \lambda_{w}^{\left( y,j \right)} \right),$ $\log\left( \lambda_{w}^{\left( y,j \right)} \right)=\beta_{0}+\beta_{1}\sin\left( \Theta_{w} \right)+\beta_{2}\cos\left( \Theta_{w} \right)+$ $+ \beta_{3}\sin\left( {\Theta_{w}}/2 \right)+\beta_{4}\cos\left( {\Theta_{w}}/2 \right)+\gamma^{\left( y,j \right)},$ $\Theta_{w}=2\cdot\pi\cdot w/{52.1775}, y=\left\{ 2014\ldots2020 \right\}.$ |  |
| --- | --- | --- |

where $\gamma^{\left( y,j \right)}$ is a year- and jurisdiction-specific random effect, cosine and sine terms describe the seasonality effect. We did not perform the adjustment for the reporting delay because of historical data covering the time period from 2014 through March 2020. We also did not adjust the time series to the influenza confirmed cases as it was done in [S1].

**References**

1. Weinberger DM, Chen J, Cohen T, Crawford FW, Mostashari F, Olson D, et al. Estimation of excess deaths associated with the COVID-19 pandemic in the United States, March to May 2020. JAMA Int Med. 2020;180(10):1336–44. (doi:[10.1001/jamainternmed.2020.3391](http://dx.doi.org/10.1001/jamainternmed.2020.3391))
